# Minimal Hepatic Encephalopathy: Effect of *H. pylori* infection and small intestinal bacterial overgrowth treatment on clinical outcomes

**DOI:** 10.1038/s41598-020-67171-7

**Published:** 2020-06-22

**Authors:** Shahab Abid, Muhammad Kamran, Adeel Abid, Nazish Butt, Safia Awan, Zaigham Abbas

**Affiliations:** 1 Section of Gastroenterology, Department of Medicine, Aga Khan University, Karachi, Pakistan; 2 Medical College, Aga Khan University, Karachi, Pakistan; 3 Department of Medicine, Aga Khan University, Karachi, Pakistan

**Keywords:** Gastroenterology, Liver cirrhosis, Liver cirrhosis, Gastroenterology

## Abstract

The effect Helicobacter pylori (Hp) infection and small intestinal bacterial over growth (SIBO) in minimal hepatic encephalopathy (MHE) is not well understood. The aim of the study was to determine the effect of eradication of Hp infection and SIBO treatment on MHE in patients with cirrhosis. Patients with cirrhosis were enrolled and MHE was determined by psychometric tests and critical flicker frequency analysis. Hp infection and SIBO were assessed by urea breath and Hydrogen breath tests respectively in patients with cirrhosis and in healthy volunteers. Patients with Hp infection and SIBO were given appropriate treatment. At six weeks follow-up, presence of Hp infection, SIBO and MHE status was reassessed. Ninety patients with cirrhosis and equal number of healthy controls were included. 55 (61.1%) patients in the cirrhotic group were diagnosed to have underlying MHE. Among cirrhotic group, Hp infection was present in 28 with MHE (50.9%) vs. in 15 without MHE (42.8%) (p = 0.45). Similarly, SIBO was present in 17 (30.9%) vs. 11 (31.4%) (p = 0.95) in patients with and without MHE respectively. In comparison with healthy controls, patients with cirrhosis were more frequently harboring Hp and SIBO (47.7% vs. 17.7% (p < 0.001) and 31.1% vs. 4.4% (p < 0.001) respectively. On follow-up, all patients showed evidence of eradication of Hp and SIBO infection. Treatment of SIBO significantly improved the state of MHE in cirrhotics, however eradication of Hp infection did not improve MHE significantly. Additionally, patients with low Model for End-Stage Liver Disease (MELD) score and belonging to Child class B had significantly better improvement in MHE. A large number of patients with cirrhosis had either active Hp infection or SIBO with or without MHE, compared to healthy controls. Treatment of SIBO significantly improved MHE in patients with cirrhosis, whereas eradication of Hp did not affect the outcome of MHE in these patients.

## Introduction

Hepatic encephalopathy (HE) is a frequent complication of chronic liver disease (CLD)^1^. HE can be precipitated by various factors like gastrointestinal bleeding, sepsis, azotemia, drugs (e.g. sedatives, diuretics), electrolyte imbalance and constipation. The most relevant substance considered in the pathogenesis of HE is ammonia, although the exact mechanisms of its neurotoxic effects are still under study^2^.

A substantial number of patients with advanced CLD have minimal hepatic encephalopathy (MHE), which is the earliest stage in the spectrum of HE. One study reported the occurrence of MHE to be as high as 50%in patients with CLD^3^. MHE, by definition, has no obvious clinical manifestations and is characterized by neurocognitive impairment in attention, vigilance and integrative function^4^. It develops in patients with significant liver function impairment or with porto-systemic shunting. MHE is associated not only with impaired daily functioning and quality of life, but is also considered as an occupational and public health hazard i.e. patient may be unfit to drive a car, operate a machinery, handle finances etc.^5^. However, current practice guidelines do not recommend treating MHE routinely^6^. MHE is not noticeable on clinical examination, but can be detected by various neuropsychological evaluations like Psychometric Hepatic Encephalopathy Score (PHES) and neurophysiological tests such as electro-encephalogram (EEG), spectral EEG, Critical Flicker Frequency (CFF), evoked potentials and computerized tests.

Various studies elucidating the relationship between active *Helicobacter pylori* (Hp) infection and hepatic encephalopathy have been published^1,7^. However, scanty data is available with regards to the same in MHE^8^.Work done by Schulz and his colleagues in a prospective clinical trial (though without healthy control group for comparison) failed to show an increased prevalence of Hp infection in patients presenting with MHE as opposed to those without MHE^9^. In addition to this, much interest has recently developed to define the role of small intestinal bacterial overgrowth (SIBO) among patients with MHE^10,11^. However, evidence of such a role in our patient population still seems to be lacking.

*H. pylori* are non-invasive, gram-negative bacteria recognized as a pathogen of upper gastrointestinal diseases, such as acute and chronic gastritis, gastro-duodenal ulcers and mucosa-associated lymphoid tissue (MALT) lymphoma. Additionally, *H. pylori* has also been characterized as group 1 (definitive) carcinogen by the International Agency for Research on Cancer (IARC) because of its association with gastric adenocarcinoma^12^. Studies on animal models have shown a significant increase in the liver fibrotic score and aminotransferase activity (and hence promote the development of CLD) in a group inoculated with Hp and CCl_4_ (carbon tetrachloride) compared with a CCl_4_treated group^13^. Moreover, by producing large amounts of highly active enzyme urease, Hp can convert urea to ammonia. This phenomenon results in alteration of neurotransmission, and hence affects consciousness and behavior, which has been implicated in causation of HE in patients with liver cirrhosis^1^.

SIBO is described as a proliferation of the bacterial population in the small intestine, particularly distal gut. CLD is associated with reduced gut motility and decreased gastric acid secretion in the stomach, resulting in gastric enteropathy. Both these factors can predispose to development of SIBO^14^. In addition, SIBO may facilitate translocation of bacteria or bacterial components (antigens) across the intestinal barrier, with harmful consequences to health, one of which could be HE^15^.

The aim of our study was to determine whether treatment of active Hp infection and SIBO play a role in improving MHE in cirrhotic patients.

## Methods

### Study design

This was a prospective cohort study.

### Setting

The study was conducted in the outpatient department of the Aga Khan University Hospital, Karachi, Pakistan.

### Sample size

It is estimated that about 55% of patients with cirrhosis would have MHE based on a positive result for both Psychometric Hepatic Encephalopathy Scoring (PHES) and Critical Flicker Frequency (CFF) analysis^16^. Assuming the CFF to have a sensitivity of 87% and specificity of 82% for MHE, and a confidence interval of 5%, the estimated sample size for this study was calculated to be 164^17^. To achieve the study objectives, the sample size was inflated by 10% to 180.

### Sampling technique

Non-probability convenient sampling was used to identify the subjects.

### Patients selection

#### Inclusion criteria

Patients with liver cirrhosis (irrespective of cause), 18 years and above, and without prior history of overt hepatic encephalopathy were included in the study.

#### Exclusion criteria

Patients currently receiving Hp eradication therapy, those on antibiotics for spontaneous bacterial peritonitis (SBP) prophylaxis or any other infection within last 4 weeks, or those who were on sedatives, rifaximin and/or lactulose (within 1 week) were excluded. Patients who had overt encephalopathy, severe cardiac, pulmonary, renal or cerebral disease, as well as those who had a history of recent upper gastrointestinal (GI) bleed (in last 6 weeks) were excluded from the study.

### Ethical clearance

Ethical approval was obtained from the Ethical Review Committee (ERC) of the Aga Khan University, Karachi, Pakistan (ERC Reference #: 2873-Med-ERC-13). Written informed consent was obtained from all participants. Patients’ identification remained anonymous throughout the study. Patients were informed that the data will be used for research purpose and publication without revealing individual identification and information. Study was performed in accordance with the principles of good clinical practice from the Declaration of Helsinki.

### Data collection procedure

All patients already diagnosed to have cirrhosis based on clinical signs and symptoms, laboratory parameters and imaging studies, meeting the inclusion criteria and agreeing to participate, were enrolled after written informed consent. The informed consent was translated in Urdu (native) language as well. Child Turcotte Pugh (CTP) and Model of End-stage Liver Disease (MELD) scores of individual patients were calculated in order to determine disease severity. These patients then underwent psychometric hepatic encephalopathy scoring (PHES) and critical flicker frequency (CFF) analysis to detect MHE. For the study purpose, a patient was diagnosed as having MHE if he tested positive for both CFF analysis and PHES testing. CFF analysis was incorporated as the examination is reliable, simple, easy to apply and can be performed without difficulty by patients with low educational background, such as patients from a country with relatively low levels of literacy.

### Diagnosis of MHE

#### Psychometric hepatic encephalopathy scoring (PHES)

PHES is a battery of neuropsychological tests which has long been regarded as a ‘gold standard’ for the assessment of MHE^18,19^. Patients were classified as having MHE when the PHES score was less than −4 points. PHES comprises of five components:^4^

#### Number connection test A

The patient was instructed to join numbered circles in order on a piece of paper. The time required to complete the task was scored.

#### Number connection test B

The patient was instructed to join numbered circles and alphabets e.g. 1, A, 2, B and so on.

#### Line tracing (trail drawing test)

The patient was asked to trace a path, 5 mm wide, as fast as possible without touching the borders.

#### Serial dotting test

The patient was asked to dot the center of circles on a piece of paper.

#### Digit symbol test

The patient was asked to learn a code in which a digit is represented by a symbol. He/she then had to reproduce the symbol corresponding to the digit.

For calculation of PHES, individuals are supposed to complete all five components.

### Critical flicker frequency (CFF) testing

The neurophysiologic CFF analysis is a tool that measures the ability of the central nervous system to detect flickering light, which is directly influenced by cortical activity^5,20,21^. In this method, an intermittent light stimulus appears as a flicker which is dependent on the frequency of light pulse presentation. The rate at which flicker just disappears is termed the critical flicker frequency (CFF). Patients were provided a CFF analyzer (a pair of spectacles shielding against outside light), and they were asked to concentrate on a red light, which was initially flickering. The frequency of the light was then gradually decreased by the operator until the patients perceived it as flickering, and they had to press a handgrip button when this happened. A lower level of 38 Hz was used as a cut-off for impaired CFF (and hence positive MHE).

### Diagnosis of active *H. pylori* infection and SIBO

Diagnosis of active H. pylori infection was made by urea breath test (UBT) using Carbon 13^22^. Patients were asked to swallow 50 mg capsule of 13 C-urea. Breath samples were collected by exhaling into a 200 ml gas storage bag to be analyzed by an infrared spectrometer, and this was performed before and 15 minutes after the consumption of capsule. For the diagnosis of SIBO, hydrogen breath test (HBT) was utilized, in which 50 gram of lactulose was given to the subject, and an increase of ≥20 parts per million (p.p.m) in hydrogen production at 90 min from base-line was taken as a positive diagnosis for SIBO.

### Treatment of active *H. pylori* infection and SIBO

In our study, all patients were subjected to UBT and HBT. Patients who were tested positive were given appropriate therapy. Triple medicine regimen which includes clarithromycin, amoxicillin (for 10 days) and a proton pump inhibitor (for another 4 weeks) was given for the eradication of H. pylori infection^23^. We chose the triple regime as it is easier to administer and because low recurrence rates of H. pylori infection (despite considerable resistance rates of approximately 36%) have been reported from our part of the world^24^.

With regards to treatment of patients with SIBO, rifaximin 1200 mg/day for 1 week, which has shown good efficacy for SIBO in various studies, was administered^25,26^. This dosage was selected as there had been substantial variation in the dose and duration of rifaximin at the time this study was conducted^27^. UBT and HBT were repeated after 6 weeks of completion of therapy^28^. CFF analysis and PHES were also repeated at the same time (i.e. after 6 weeks of completion of triple therapy and SIBO treatment) to detect an improvement in MHE. Rifaxamin was given to patients who came out as SIBO positive. Of note, none of the patients were given rifaximin as prophylaxis for hepatic encephalopathy during the follow-up period.

**Figure 1:**
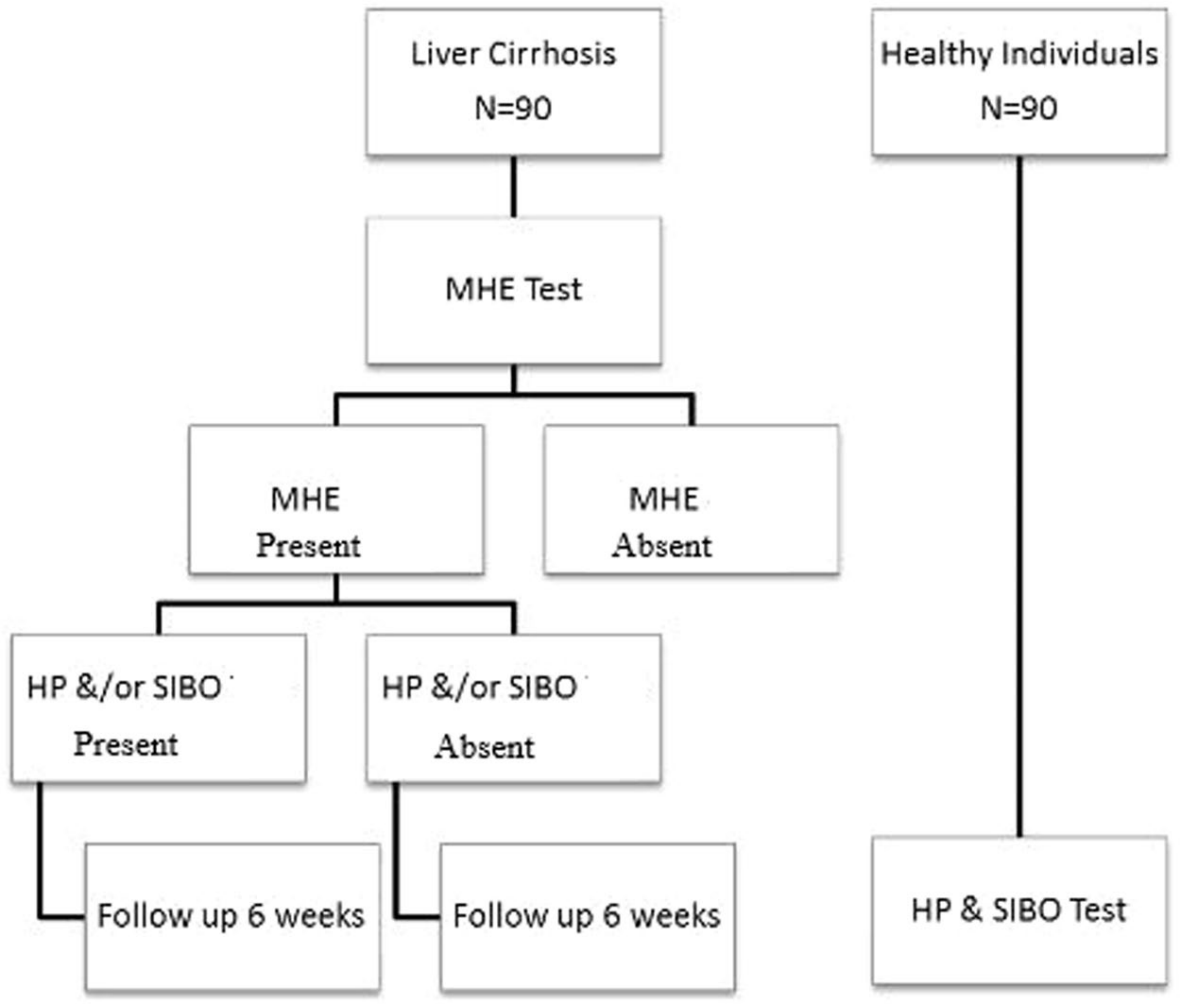
Study Methodology- Flowchart. HP: Helicobacter pylori. SIBO: Small Intestinal Bacterial Overgrowth. MHE: Minimal Hepatic Encephalopathy.

For comparison with healthy subjects, 90 age and gender-matched controls (e.g. patients’ relatives, healthy hospital employees, etc.) were also enrolled in the study after written informed consent and underwent UBT and HBT. Those who were found positive for Hp and SIBO were treated accordingly (Fig. 1).

### Statistical analysis

Data analysis was done using IBM SPSS Statistics for Windows, version 19.0 (Armonk, NY: IBM Corp. IBM Corp. Released 2011). Initially, the frequency was generated for all the variables. Continuous variables were presented as mean ± SD and categorical variables were presented as a percentage. Continuous variables between groups were compared by using unpaired t test or Mann Whitney test where appropriate. Categorical variables were compared by using the chi-square test or Fisher exact test. A p-value <0.05 was considered significant.

## Results

A total of 90 patients were included in the study. Mean age was 44.9 ± 11.6 years, and the majority were males (53.3%). The clinical and laboratory parameters of cirrhotic patients with and without MHE are shown in Table 1.

**Table 1:** Characteristics of patients with or without MHE^Ω^.

	With MHEn = 55 (%)	Without MHEn = 35 (%)
Age (years)	44.6 ± 11.9	45.5 ± 11.8
Gender		
Male	29(52.7)	19(54.3)
Female	26(47.3)	16(45.7)
Ascites		
None	35(63.6)	26(74.3)
Mild to Moderate	19(34.5)	9(25.7)
Severe	1(1.8)	0
Concomitant HCC^µ^	4(7.3)	0
Hemoglobin (g/dl)	10.3 ± 1.6	10.1 ± 1.7
Platelets (x10E9/L)	92.6 ± 34	103.7 ± 38.1
Total Bilirubin (mg/dl)	2.6 ± 0.66	2.5 ± 0.78
Direct Bilirubin (mg/dl)	1.5 ± 0.53	1.4 ± 0.61
Indirect Bilirubin (mg/dl)	1.1 ± 0.44	1.04 ± 0.46
Albumin (g/dl)	2.7 ± 0.32	2.8 ± 0.39
Prothrombin time (sec)	15.4 ± 3.0	15.4 ± 2.5
INR^∞^	1.4 ± 0.28	1.3 ± 0.25
Creatinine (mg/dl)	1.05 ± 0.22	1.02 ± 0.22
CTP^α^ score	8.5 ± 0.99	8.05 ± 0.83*
MELD^β^ score	14.4 ± 3.06	13.8 ± 3.1

^*^p < 0.05.

^Ω^MHE: Minimal Hepatic Encephalopathy.

^µ^HCC: hepatocellular carcinoma,

^∞^INR: International normalization ratio.

^α^CTP: Child-Turcotte-Pugh,

^β^MELD: Model for End-Stage Liver Disease.

Out of 90 cirrhotic patients, 55 (61.1%) were diagnosed to have underlying MHE based on a positive CFF analysis and PHES. Patients with MHE had a significantly higher CTP score than those without MHE (p = 0.02). Among patients who had MHE, 28 (50.9%) were positive for UBT (representing active Hp infection) as opposed to 15 (42.8%) in the non-MHE group (p = 0.45). Similarly, in the MHE group, 17 (30.9%) patients were diagnosed to have SIBO (based on a positive HBT), while 11 (31.4%) were SIBO positive in the non-MHE group (p = 0.95).

In comparison with healthy controls, overall, 43/90 (47.7%) patients with cirrhosis were positive on UBT as compared to 16/90 (17.7%) in age and gender-matched controls (p < 0.001). Likewise, there were 28/90 (31.1%) patients with cirrhosis who were positive on HBT, as opposed to only 4/90 (4.4%) positive among controls (p < 0.001) (Table 2).

**Table 2:** Frequency of active Hp* infection and SIBO^Ω^ in patients and controls.

	Healthy Controlsn = 90	Patients with liver cirrhosis		
		MHE^∞^ present Patientsn = 55	MHE absent Patientsn = 35	*p* value
SIBO present	4 (4.4)	17 (30.9)	11 (31.4)	<0.001
SIBO absent	86 (95.6)	38 (69.1)	24 (68.6)	
*Hp infection present*	16 (17.7)	28 (50.9)	15 (42.9)	<0.001
*Hp infection absent*	74 (93.3)	27 (49.1)	20 (57.1)	

^*^Hp: Helicobacter pylori

^Ω^SIBO: Small Intestinal Bacterial Overgrowth

^∞^MHE: Minimal Hepatic Encephalopathy.

All subjects received appropriate therapy for active Hp and SIBO (as mentioned earlier). Six weeks after therapy, UBT and HBT were repeated in all patients irrespective of their baseline status, and interestingly those individuals who initially tested positive on either UBT and/ or HBT were now found to be negative on repeat testing, indicating complete eradication of both the conditions. Seven cirrhotic patients who had underlying MHE remained positive for it even after treatment with anti-Hp therapy and/ or rifaximin. Among such patients, 6 had active Hp infection, whereas 1 patient had SIBO. Six of the 12 individual patients with MHE, who did not have either active Hp infection or SIBO, were still found to have MHE at 6 weeks follow-up testing.

The overall improvement in MHE among patients who initially had SIBO versus those who did not have SIBO was found to be statistically significant. However, MHE among patients who had Hp infection did not show any significant improvement in their MHE. Furthermore, the improvement in the level of MHE was more prominent in patients with relatively less advanced liver disease i.e. those with lower MELD score and belonging to Child-Turcotte-Pugh (CTP) class B (Table 3).

**Table 3:** Outcome of MHE^∞^ after six weeks of treatment of active Hp* infection and SIBO^Ω.^.

Cirrhotic patients with MHE n = 55	MHE improved	MHE not improved	*p* value
SIBO positive	16 (38.1)	1 (7.7)	0.03
SIBO negative	26 (61.9)	12 (92.3)	
*Hp* positive	22 (52.4)	6 (46.2)	0.69
*Hp* negative	20 (47.6)	7 (53.8)	
Meld score	14.02 ± 2.80	16.0 ± 3.46	0.04
CTP classBC	40 (95.2) 2(4.8)	8 (61.5) 5 (38.5)	0.001

^∞^MHE: Minimal Hepatic Encephalopathy

^*^Hp: Helicobacter pylori

^π^SIBO: Small Intestine Bacterial Overgrowth.

## Discussion

It is a well-known fact that SIBO is common in patients with liver cirrhosis^29^, which most likely occurs as a result of delayed small bowel transit in such individuals. Our study elucidated a significant improvement in the state of MHE in cirrhotic patients after treatment with SIBO therapy compared to those who had MHE without SIBO. Similar observations were also noted in a previously published study in which 26 out of 60 patients with cirrhosis who had MHE were treated by rifixamin for one week and found a significant improvement in MHE after treatment^10^.

On the other hand, we could not extrapolate the same results of improvement in MHE with regards to those patients with MHE who had Hp infection compared to those who have no Hp infection. Effect of Hp infection eradication over MHE in patients with cirrhosis remained debatable and earlier studies have also demonstrated controversial results; one such study has shown that anti-Hp therapy results in reducing ammonia levels in blood and hence improvement in MHE^8^. On the contrary, another study showed that Hp eradication does not induce any improvement in the psychometric and/or electrophysiological tests, which are used to diagnose MHE^30^. One plausible explanation for the above phenomenon is the fact that PPI use (given as part of Hp eradication regimen) has itself been linked to development of MHE, by inducing changes in the gut flora and subsequently leading to increased ammonia production^31^. We also use PPI in our study for an extended period of 4 week after triple therapy. Therefore beneficial effect of eradication of Hp infection over MHE in present study is not evident hence we do not suggest treatment and eradication of Hp solely for improvement in MHE.

Our study showed that the frequency of Hp and SIBO was considerably higher in cirrhotic patients as compared to the healthy controls. This observation has been elucidated by previous studies as well^32,33^. An Italian study^34^ demonstrated Hp antibodies to be positive in 89% of cirrhotic patients as compared to 59% controls (p < 0.0001). Later, in another study it was discovered that Hp infection along with an elevated transforming growth factor- β1 (TGF-β1) may accelerate hepatic fibrosis through increased TGF-β1-induced pro-inflammatory signaling pathways in hepatic stellate cells^35^. A recent study also found Hp infection to be more frequent among patients with cirrhosis secondary to chronic viral hepatitis, which happens to be the most prevalent cause of cirrhosis in our part of the world^36^. However, the cause and effect relationship between Hp infection and cirrhosis is still a matter of dispute.

Our study had a few limitations. Firstly, we did not measure serum ammonia levels of the subjects, as was performed in earlier similarly conducted studies on MHE^16^. However, as previous work has shown that arterial ammonia does not seem to play a major role in the diagnosis of MHE, we think that performing this test will not contribute substantial information^37^. The diagnosis of SIBO in our patients was not based on jejunal aspiration and culture, which is considered the gold standard. This method is invasive, as it requires small bowel intubation and laboratory skills in isolating anaerobes. Therefore, the inexpensive and non-invasive HBT has been utilized as has been done previously^38^ as a sole diagnostic tool for SIBO.

## Conclusion

Compared to healthy controls a significantly large number of cirrhotic patients had either active Hp infection or SIBO. Treatment of SIBO improves MHE in patients with cirrhosis, while eradication of Hp infection was not associated with improvement in MHE. Moreover, significant improvement in MHE was evident in patients with low MELD scores and belonging to CTP class B as compared to those with higher MELD scores and CTP class C respectively.

We suggest to treat SIBO in cirrhotic patients with MHE, however Hp eradication is not beneficial in such patients.
